# Patient-reported outcome data generated in a clinical intervention in community mental health care - psychometric properties

**DOI:** 10.1186/1471-244X-12-113

**Published:** 2012-08-17

**Authors:** Stefan Priebe, Eoin Golden, Rosemarie McCabe, Ulrich Reininghaus

**Affiliations:** 1 Queen Mary University of London, London, UK; 2 College London, Institute of Psychiatry, De Crespigny Park, London, SE5 8AF, UK; 3 Unit for Social and Community Psychiatry, Barts and the London School of Medicine and Dentistry, Newham Centre for Mental Health, Queen Mary University of London, London, E13 8SP, UK

## Abstract

**Background:**

DIALOG is an intervention to structure the communication between patient and key worker, which has been shown to improve patient outcomes in community mental health care. As part of the intervention, patients provide ratings of their subjective quality of life (SQOL) on eight Likert type items and their treatment satisfaction on three such items. This study explored the psychometric qualities of the outcome data generated in the DIALOG intervention to explore whether they may be used for evaluating treatment outcomes.

**Method:**

Data were taken from 271 patients who received the DIALOG intervention. All patients were diagnosed with schizophrenia or a related disorder and treated in community mental health care. For SQOL and treatment satisfaction as assessed in the DIALOG intervention, we established the internal consistency (Cronbach’s alpha), the convergent validity of SQOL items (correlation with Manchester Short Assessment of Quality of Life [MANSA]) and treatment satisfaction items (correlation with Client Satisfaction Questionnaire [CSQ]), the concurrent validity (correlations with Positive and Negative Syndrome Scale [PANSS]) and the sensitivity to change (t-test comparing ratings of the first and last intervention). We also explored the factorial structure of the eight SQOL items.

**Results:**

The internal consistency of the eight SQOL items was .71 and of the three treatment satisfaction items .57. SQOL scores were correlated with the MANSA (r = .95) and PANSS scores (general psychopathology: r = −.37, positive symptoms: r = −.27, negative symptoms: r = −.27). Treatment satisfaction scores were correlated with the CSQ (r = 0.36) and the PANSS (r = −.29, -.20, -.20). SQOL and treatment satisfaction score improved significantly over time. SQOL items loaded on two meaningful factors, one capturing health and personal safety and one reflecting other life domains.

**Conclusions:**

The psychometric qualities of the SQOL scores generated in DIALOG are strong. The properties of the three treatment satisfaction items may be seen as acceptable. Although DIALOG has been designed as a therapeutic intervention, it can generate outcome data on SQOL and treatment satisfaction with acceptable psychometric qualities.

## Background

The importance of routine outcome assessment, whereby patient-reported outcomes (PROs) are regularly collected in mental health services, has been increasingly emphasised in recent years. Research on the development and evaluation of PROs has markedly increased over the past twenty years [1]. In the United Kingdom, national patient organisations have emphasised the importance of measuring PROs [2,3], while service providers are expected to use PROs for assessing the quality of routine care [4,5]. Subjective quality of life (SQOL) and treatment satisfaction are particularly common PROs [6-8] and have been frequently used in the evaluation of community treatments for patients with psychosis.

Routine assessment of PROs has been recommended for different reasons [9]. Treatment-level data can inform the care provided to individual patients. Service level data can encourage reflective practice of clinicians, provide transparency about the outcomes in a given service and feed into the evaluation and quality management. Large-scale pooling of the data can help to establish whether national targets relating to mental health have been met, and inform regional and national funding of mental health services.

Despite these potential advantages, routine assessments of PROs are not widely implemented [10]. Explanations for this include the time-consuming nature of collecting outcomes, and the perception of data collection as a burden [11]. Patients can suffer from ‘survey fatigue’ and be reluctant to fill in questionnaires when the data is of no immediate use to their treatment.

An alternative approach for collecting PROs may be presented by the DIALOG intervention [12]. This computer-mediated intervention structures part of the patient-clinician communication in community mental health care. Clinicians ask patients how satisfied they are with eight life domains and three treatment aspects. Patients rate their satisfaction on Likert type scales and the ratings are intended to inform the therapeutic dialogue between clinician and patient. The procedure allows for a comprehensive assessment of patients’ satisfaction and facilitates a patient centred discussion. In a randomised controlled trial in six European countries the use of DIALOG every two months for one year was associated with a better quality of life, fewer unmet needs and higher treatment satisfaction. Current research aims to further refine DIALOG and test its effectiveness in general adult and forensic psychiatric services. Randomised controlled trials using DIALOG as part of novel therapeutic interventions are being conducted in the Netherlands, the United Kingdom and the United States.

Whilst DIALOG in these studies is always used as a therapeutic intervention to improve outcomes, the question arises as to whether the data generated in the intervention are valid PROs and can be used outside the intervention for assessing treatment outcomes. This would allow the assessment of PROs in a clinically meaningful intervention that can be routinely administered, has no additional burden on the patient and does not require the service to arrange separate interviews or surveys.

For using the data generated in the intervention as outcome measures, the psychometric properties need to be established. Evidence is particularly required to test whether the small number of items in the DIALOG intervention is still sufficient to provide reliable findings that are sensitive to change.

The aim of this study is to assess the psychometric properties of the eight items on SQOL and the three items on treatment satisfaction. For SQOL and treatment satisfaction we established the internal consistency, the convergent validity with established scales, the concurrent validity with symptom levels, and the sensitivity to change. For assessing the structural validity of the SQOL items we explored their factorial structure.

## Method

### Sample and material

Data were taken from the intervention group in the previously mentioned DIALOG trial, i.e. from all 271 patients who were randomly allocated to receiving the intervention. Patients were recruited in community mental health services in Granada (Spain), Groningen (The Netherlands), London (United Kingdom), Lund (Sweden), Mannheim (Germany), and Zurich (Switzerland). Of these patients, 88 (32.5%) were women. The mean age was 42.5 (SD = 11.3) years. All of them had a diagnosis of schizophrenia or a related disorder (F 20–29) according to ICD-10 [13]. The mean length of illness was 16.6 (SD = 10.5) years. The trial was pragmatic, had wide inclusion criteria and compared the regular use of the DIALOG intervention (about every two months) in addition to treatment as usual with treatment as usual without any additional intervention. Treatment was provided in community mental health services and the study period was 12 months. Written informed consent was obtained from patients for participation in the study and publication of the results. The trial, sample and context have been described in more detail in previous publications [12,14].

The SQOL component of the DIALOG intervention consists of eight items – mental health, physical health, job situation, accommodation, friendships, leisure activities, partner/family, and personal safety – rated by the patient on a Likert scale ranging from 1 (=couldn’t be worse) to 7 (=couldn’t be better). The treatment satisfaction component of the DIALOG intervention consists of three items – medication, practical help received and meetings with mental health professionals – rated on the same scale. The Manchester Short Assessment of Quality of Life (MANSA) [15,16] contains 12 items measuring SQOL. These include the eight SQOL items of DIALOG, as well as satisfaction with life as a whole, financial situation, people one is living with, and sex life. The Client Satisfaction Questionnaire (CSQ) [17] assesses satisfaction with treatment and consists of eight items rated from 1 to 4, with higher scores indicating greater treatment satisfaction. The Positive and Negative Syndrome Scale (PANSS) [18] assesses positive, negative and general symptoms and consists of 30 items rated on a scale of 1 to 7, with higher scores indicating more severe symptoms.

### Statistical analysis

In comparing DIALOG with other scales (PANSS, MANSA, CSQ) and identifying the factorial structure, data of the baseline assessment and the first DIALOG intervention were used. For the assessment of the internal consistency and sensitivity to change, assessments of the last DIALOG intervention in the study were also considered. The period of time between first and last intervention ranged from seven to 12 months. Since the number of assessments per patient and the time of the assessments varied substantially in the pragmatic trial, data generated in interventions other than the first and last one were not considered in the analyses of this paper.

### Reliability

As a measure of reliability we assessed the internal consistency of the eight SQOL items and the three treatment satisfaction items in DIALOG by computing Cronbach’s alpha.

### Convergent and concurrent validity

For assessing the convergent and concurrent validity of the SQOL and treatment satisfaction scores, we established Spearman’s correlations of SQOL scores with the MANSA scores, of the treatment satisfaction scores with the CSQ scores, and of both SQOL and treatment satisfaction scores with symptom scores on the PANSS.

### Sensitivity to change

For assessing sensitivity to change we compared ratings in the first and last intervention using t-tests for dependent samples.

### Structural validity

We established the factorial structure of the eight SQOL items of DIALOG. Exploratory factor analysis for ordinal data was conducted [19] on both sets of items. Model estimation used the robust weighted least squares means and variance adjusted (WLSMV) estimator in MPlus, version 5.2 [19]. The optimum number of factors was determined by computing the root mean square error of approximation (RMSEA) [20], the Comparative Fit Index [21], and the Tucker Lewis Index [22]. A good model fit is generally indicated by a low RMSEA (<0.10 for acceptable and <0.05 for very good fit) [23], and a high CFI and TLI (>.090 for acceptable and >0.95 for very good fit) [20,24].

For the treatment satisfaction scores in DIALOG, we did not test the structural validity. Evidence suggests that treatment satisfaction in mental health is a rather global and coherent construct, which is one of the reasons for addressing treatment satisfaction in DIALOG with only three items [8,25,26].

## Results

### Scores of SQOL, treatment satisfaction and symptoms

All patients in the intervention group participated in the DIALOG intervention and generated outcome data. The average mean score of the eight SQOL items in DIALOG was 4.83 (SD = .91), and of the three treatment satisfaction items 5.49 (SD = .94).

The average mean score of the MANSA was 4.73 (SD = .87) and the mean of the CSQ sum score was 25.76 (SD = 4.09). The means of the PANSS positive, negative, and general symptom scores were 15.0 (SD = 5.8), 17.2 (SD = 7.0), and 32.6 (SD = 10.1), respectively.

### Sensitivity to change

At the last intervention, the average mean score of the eight SQOL items was 5.39 (SD = .85), and of the three treatment satisfaction items 5.93 (SD = .75). The improvements for both SQOL and treatment satisfaction were statistically significant (p <0.001).

### Internal consistency

Regarding DIALOG, Cronbach’s alpha was .71 for the eight SQOL items (.78 at the last DIALOG intervention) and .57 for the three treatment items (.53 at the last DIALOG intervention).

### Convergent validity

Spearman’s rank correlation coefficient between the MANSA mean score and DIALOG life satisfaction mean score was r = .94 (p <0.001). The DIALOG treatment satisfaction mean score and CSQ sum score were positively correlated (r = .33, p <.001).

### Concurrent validity

The mean score of the SQOL items in DIALOG was negatively correlated with the PANSS general (r = −.37, p <.001), positive (r = −.27, p <.001), and negative (r = −.27, p <.001) symptom scores. The DIALOG treatment satisfaction mean score was negatively correlated with the PANSS general (r = −.29, p <.001), positive (r = −.20, p <.01), and negative (r = −.20, p <.01) symptom scores.

### Structural validity

In terms of the SQOL items in DIALOG, Table 1 shows model fit statistics for 1- and 2-factor solutions yielded by exploratory factor analysis. The 2-factor solution provided the best model fit. Table 2 displays the factor loadings of the 2-factor solution. Satisfaction with physical health, mental health and personal safety loaded on one factor, whilst satisfaction with job situation, friendships, leisure activities, accommodation, and partner/family loaded on the second factor. All loadings other than that of job situation on the second factor (.38) were higher than .40.

**Table 1:** Model fit statistics for the 1- and 2- factor solutions for the eight SQOL items in DIALOG

	**χ**^**2**^	**CFI**	**TLI**	**RMSEA**
Factor solutions				
1-factor solution	84.8	.91	.88	.11
2-factor solution	36.4	.97	.93	.08

*Note:* χ^2^, model chi-square; CFI, Comparative Fit Index; TLI, Tucker Lewis Index; RMSEA, Root Mean Squared.

Error of Approximation; AIC, Akaike Information Criterion; BIC, Bayesian Information Criterion.

**Table 2:** Eight DIALOG SQOL items, factors, and factor loadings (confidence intervals) for the 2-factor solution

	**Factor 1**	**Factor 2**
***Satisfaction with***		
Job situation	.18 (.02 to .35)	.38 (.22 to .53)
Friendships	-.17 (−.36 to .03)	.71 (.54 to .88)
Leisure activities	.02 (−.05 to .08)	.73 (.62 to .83)
Accommodation	-.05 (−.20 to .10)	.41 (.27 to .56)
Personal safety	.49 (.31 to .67)	.25 (.07 to .43)
Partner/family	.03 (−.18 to .24)	.48 (.30 to .66)
Physical health	.70 (.58 to .82)	-.01 (−.02 to .01)
Mental health	.52 (.33 to .71)	.20 (.01 to .39)

## Discussion

For the eight items measuring SQOL in the DIALOG intervention, the study identified a satisfactory internal consistency, a very high convergent validity with scores on the MANSA, and a meaningful factorial structure. One factor captured mental and physical health as well as personal safety, whilst the other factor comprised satisfaction with all social areas of life. The lowest loading of any item was for satisfaction with job situation, which is a particular domain in this patient group since most patients are without regular employment. For the three treatment satisfaction items, the internal consistency was substantially lower and the convergence with the CSQ scores was moderate. For both SQOL and treatment satisfaction scores, the correlations with symptom levels were plausible and in the expected direction and range [27]. Longitudinal data were obtained within the intervention arm of a randomised controlled trial in which DIALOG was repeatedly used. Over a period of up to a year, SQOL and treatment satisfaction scores showed significant improvements indicating that they are sensitive to change.

The study did not assess the construct validity of the SQOL and treatment satisfaction scores in DIALOG. The face validity may be regarded as high which is essential for the intervention: only if the patients and clinicians regard the items as important and relevant, are they likely to use the intervention routinely and regularly for structuring their communication.

A recent review of PROs in patients with psychosis has suggested that the validity of scales with satisfaction items is based on more evidence than assessment methods using other types of questions [28]. All 11 items used in the DIALOG intervention are satisfaction ratings and therefore may be seen as using the best evaluated approach for assessing PROs in this patient group.

To what extent do the findings of this study justify the use of the scores that are generated in the DIALOG intervention, i.e. in a meeting with a clinician that has a therapeutic purpose, as psychometrically acceptable outcome data? The eight SQOL items appear to provide a valid measure of SQOL. The items are identical with items used in the MANSA, and the analysis in this study shows that the reduction of 12 SQOL items in the MANSA to eight in the DIALOG intervention does not compromise the psychometric properties.

For treatment satisfaction, the findings are less clear. Three is the minimum number of items constituting a scale and, by definition, the internal consistency of scales decreases with fewer items. Also, for the therapeutic purpose of the DIALOG intervention, the items are designed to cover distinct areas of treatment, which further compromises the internal consistency of a three-item scale. In light of this, the internal consistency found in this paper may be seen as reasonable and sufficient for using the score in the evaluation of outcomes. The correlation with CSQ is moderate which may be explained by the different focus of the two scales. Whilst CSQ covers community health care in a more general sense, the three items in DIALOG address the main components of treatment that is provided in community mental health teams, i.e practical help, talks with mental health professionals and medication. Although the three items cover different treatment aspects, their mean score is sensitive to change, which is important for monitoring treatment outcomes.

The findings suggest that the SQOL and treatment satisfaction scores generated in the DIALOG intervention possess sufficient psychometric properties to be used as outcome data in the evaluation of routine community mental 'health care. Using the data of a regularly administered therapeutic intervention as outcome evaluation may be a solution to the problem of generating PROs in routine care. The obvious advantage is that neither clinicians nor patients would have to engage in a separate exercise for assessing such outcomes making the procedure most economical. Possibly even more important is that the approach overcomes the common problem of low response rates and selection biases in patient surveys. In the study, all patients who consented to participate in the trial and were allocated to the intervention generated PROs within DIALOG. Using the data of the DIALOG intervention means that outcome data is available for every single patient who participates in the intervention, which is equivalent to a response rate of 100%. The existing research evidence [12] suggests that the intervention can be used with a wide range of patients, including those with severe and persisting psychotic disorders. Thus, the approach is likely to provide PROs data with much less selection bias than separate surveys and other separate outcome assessments.

Integrating outcome assessments in clinical routine meetings raises the issue as to whether PROs should be assessed in a meeting with the clinician rather than with an independent researcher or administrator. If patients rate their satisfaction for life and treatment in the presence of their clinicians, the rating may be biased, e.g. in the direction of social desirability. However, experimental research has shown that such a bias neither consistently nor substantially influences PRO ratings in this context [29]. Moreover, if the ratings are to be used to evaluate and improve the quality of individual treatment, clinicians must be aware of them regardless; therefore, the potential rating bias can never be totally avoided.

Assessing outcome data in the DIALOG intervention is consistent with the principles for implementing routine outcome assessment in mental health services as identified in a review by Slade [9]. He concluded that standardised measures should be used, that data collection should be cheap and simple, that feedback should quick, easy and meaningful and that data should be collected longitudinally. He further emphasised the importance of minimising the time of clinicians spent on this, and of the role of technology in preserving it. DIALOG complies with all of these recommendations, and is free to use and easy to implement. It is mainly a therapeutic intervention to make the communication between patient and clinician more effective.

## Conclusions

The findings of this study suggest that the data generated within the DIALOG intervention possess sufficient psychometric properties to be used as outcome data in the evaluation of services in routine community mental health care of patients with schizophrenia and related disorders, and that this data can show significant changes over time. Further research is required to test whether this also applies to other samples and other settings.

## Competing interests

The authors declare that they have no conflicts of interest.

## Authors’ contributions

SP designed and supervised the study and drafted the manuscript. EG reviewed the relevant literature and assisted in drafting the manuscript. UR participated in the design of the study and performed the statistical analyses. RMC was a co-investigator on the DIALOG trial and advised on the drafting of the manuscript. All authors read and approved the final manuscript.

## Pre-publication history

The pre-publication history for this paper can be accessed here:

http://www.biomedcentral.com/1471-244X/12/113/prepub
